# Landscape as a Shared Space for Badgers and Cattle: Insights Into Indirect Contact and Bovine Tuberculosis Transmission Risk

**DOI:** 10.1002/ece3.71114

**Published:** 2025-04-15

**Authors:** Emma L. Holmes, Maria J. H. O’Hagan, Fraser D. Menzies, Andrew W. Byrne, Kathryn R. McBride, Charles M. McCormick, David M. Scantlebury, Neil Reid

**Affiliations:** ^1^ Institute for Global Food Security (IGFS), School of Biological Sciences Queen's University Belfast Belfast UK; ^2^ Agri‐Food and Biosciences Institute (AFBI), Veterinary Sciences Division Belfast UK; ^3^ Department of Agriculture, Environment and Rural Affairs (DAERA), Veterinary Epidemiology Unit Belfast UK; ^4^ Department of Agriculture, Food and the Marine (DAFM), One Health One Welfare Scientific Support Team Dublin 2 Ireland

**Keywords:** cattle, European badgers, grazing, indirect contact, *Meles meles*

## Abstract

Though the magnitude of effect is uncertain, badger–cattle indirect contact has been implicated in bovine tuberculosis (bTB) transmission risk to cattle despite a paucity of data on badger space use. This study tracked field use by 35 GPS‐collared bTB test‐negative badgers (*n* = 3738 locational fixes, average fixes/badger = 107) and cattle grazing regimes at 446 fields over one grazing season (May–November 2016) on 18 farms (*n* = 56,202 field‐days). Individual badger visits spanned on average 3 farms (max. 9 farms). Badgers entered fields when occupied by grazing cattle on 20% of field‐days (nights). Most individual badgers (*n* = 25; 71%) were recorded in the same field as cattle on multiple occasions (up to 124 field‐days each). There was substantial interindividual variation, with 29% of badgers (*n* = 10) never co‐occurring with cattle. Badger field use was positively associated with dairy (rather than beef) production (especially when grazing cattle were present) and with fodder and rough grazing fields (compared with improved pasture and ‘other’ cattle‐related land use). Badgers were recorded in larger fields (range 0.06 to 10.9 ha) more frequently, especially when not actively grazed. They were significantly less likely to use fields with calves compared to fields containing cattle of other age groups. The presence of a badger sett in a field increased the likelihood of field use by tracked badgers. Farm management that minimises cattle–badger indirect contact in fields with setts may reduce bTB transmission risk to cattle. Delaying grazing of fodder fields after (silage) harvest until sward length has increased, restricting grazing to improved pastures, keeping calves with cows longer, or ensuring all batches of cattle have at least some calves present and not grazing fields with badger setts (or fencing around setts to prevent cattle access) may provide simple, cost‐effective strategies to reduce indirect badger–cattle contact, thus potentially lowering bTB transmission risk.

## Graphical abstract

Although currently unproven, badger–cattle indirect contact has been implicated in the bovine tuberculosis transmission risk to cattle. In this study, we explore tracked field use by badger against cattle grazing regimes.

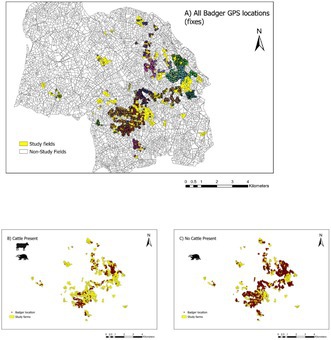

## 1 Introduction

Bovine tuberculosis (bTB) is a bacterial infection of economic importance, mainly affecting cattle. Bovine TB is predominantly caused by *Mycobacterium bovis* , which is a part of the *Mycobacterium tuberculosis* complex (More 2009; Sheridan 2011; Godfray et al. 2013; More and Good 2015). The majority of bTB lesions are found in the respiratory system of cattle, and the aerosol route is considered the main route for cattle‐to‐cattle transmission (Cassidy 2006; Menzies and Neill 2000; Skuce et al. 2012). The minimum infective dose of *M. bovis* via respiratory transmission is thought to be less than 1 cfu (colony forming unit) and occurs through the inhalation of infective droplet nuclei (Menzies and Neill 2000; Skuce et al. 2012). Ingestion is considered a much less common transmission route, as it requires very large numbers of *M. bovis* to survive the digestive process, and most alimentary lesions are thought to be caused by the swallowing of infected sputum coughed up from established lung lesions (Menzies and Neill 2000; Skuce et al. 2012).

Many countries have successfully achieved bTB official freedom using bTB programmes based on antemortem testing of the national cattle population supplemented by slaughterhouse postmortem surveillance (Rivière et al. 2014). However, bTB remains endemic in several countries in which eradication is complicated by *M. bovis* being endemic in multiple hosts, including wildlife (Broughan et al. 2016; Livingstone et al. 2015; Palmer 2013). In the UK and Ireland, the Eurasian badger ( *Meles meles* ) is widely considered to be a reservoir host of *M. bovis* , with sporadic interspecies transmission complicating bTB eradication programmes (More 2009; Godfray et al. 2013; Broughan et al. 2016). Experimental transmission between species has been demonstrated, and the application of whole genome sequencing of *M. bovis* has provided further evidence of this interspecies transmission, although intraspecies transmission is still considered to occur far more frequently (Little et al. 1982; Crispell et al. 2019; Rossi et al. 2022).

Badgers display similar *M. bovis‐*related pathological patterns to cattle, with the addition of infected bite wounds thought to be caused by territorial and dominance disputes amongst badgers (Corner et al. 2011; Courcier et al. 2018; Jenkins et al. 2008). The precise mechanisms by which interspecies transmission of *M. bovis* occurs are unknown. Studies in Northern Ireland (NI) have indicated that direct contact between species to enable animal‐to‐animal transmission is rare (O'Mahony 2014; Campbell et al. 2019; Menzies et al. 2019) but has been observed in GB studies (Garnett et al. 2002; Drewe et al. 2013; Scantlebury et al. 2004; Woodroffe et al. 2016; Judge et al. 2011). However, indirect contact has been observed at badger setts and water troughs (Campbell et al. 2019) and indirect contact through the common sharing of space may be of more importance in the route of *M. bovis* transmission (noting that no transmission mechanism has yet been elucidated). Furthermore, *M. bovis* has been shown to survive in the environment (e.g., soils) for up to several months (Rodríguez‐Hernández et al. 2016; Allen et al. 2021).

Irrespective of the mechanisms, sharing of spatial locations between cattle and badgers would be required for interspecies transmission of *M. bovis* (Woodroffe et al. 2021).

Most cattle are housed during winter, which may facilitate bTB cattle‐to‐cattle transmission due to high animal densities and limited air flow (Skuce et al. 2012; Kleinlützum et al. 2013). Rotational grazing systems are common in the UK and Ireland, resulting in cattle occupying different fields throughout the grazing season (from spring to early autumn) and moving frequently to ensure a fresh supply of grass. This could facilitate indirect contact between cattle and badgers utilising the same space but at different times, with *M. bovis* transmission possibly occurring through contaminated fomites such as soil or water (O'Mahony 2014; Campbell et al. 2019; Scantlebury et al. 2004).

When cattle are grazing, they tend to avoid consuming forage contaminated with badger faeces and urine; however, when grass availability is low, cattle may graze contaminated grass (Woodroffe et al. 2016). In regions of medium to high density badger populations (3 or more badgers per km^2^) with low woodland cover, badgers may construct their setts and latrines within field bounding hedgerows (Menzies et al. 2019; Byrne et al. 2012; O'Hagan et al. 2016). Cattle investigate hedgerow badger setts when they have access (Campbell et al. 2019) with cattle of low dominance rank potentially being more susceptible to disease (Scantlebury et al. 2006). Badgers are known to forage in fields recently harvested for silage (fodder fields) where earthworms are more easily collected after the removal of the sward. Some farmers graze cattle on the fresh regrowth in such fields, which presents a risk of contact with bTB fomites (Ward et al. 2006). In areas where badgers spend time on pasture, there is also a risk that they may encounter cattle manure contaminated with viable *M. bovis* bacilli (Phillips et al. 2003). Indeed, badgers actively forage in and around cow pats because of the invertebrates they attract, thus increasing the bTB transmission risk from cattle to badgers (Kruuk 1978). Viable bacilli have been collected from cow pats in field sites up to 15 months after deposition (Young et al. 2005), which presents a potential environmental source of infection for both cattle and badgers.

In Northern Ireland, badgers generally inhabit loose territories (Feore and Montgomery 1999; Magowan et al. 2022) within which they travel typically 1–3 km a night (O'Corry‐Crowe et al. 1993) and sometimes more than 10 km (Redpath et al. 2023). Individual bTB‐infected badgers can be aggregated within social groups, especially where there is a relatively stable social structure and geographical territory (Delahay et al. 2002). Moreover, individual badgers roaming larger‐than‐average distances may be at the highest bTB infection risk (Vicente et al. 2007) as has been described in other studies for diseases other than bTB (Fèvre et al. 2006). Identifying factors associated with badger field use on cattle farms may help inform strategies to reduce indirect contact between badgers and cattle, lowering indirect bTB transmission risk. Previous studies in Northern Ireland have shown that badger movements were not random (Magowan et al. 2022; Redpath et al. 2023; O'Hagan et al. 2021). A study using GPS collars on 105 individual badgers clearly demonstrated marked differences between the intensity of movements across each badger's territory using 50% and 95% kernel density maps (Redpath et al. 2023; O'Hagan et al. 2021). Other factors such as season and sex also impacted monthly 95% kernel density ranges (Redpath et al. 2023; O'Hagan et al. 2021). Using dead reckoning to refine badger movements also showed that, even within fields, badgers spend more time at field margins and hedgerows (Magowan et al. 2022).

The co‐occurrence of cattle with badger setts and latrines, along with the adjacency of neighbouring cattle herds, was reported previously (Campbell et al. 2020). The current study aimed to determine factors affecting badger field use in relation to grazing cattle by tracking field use by GPS‐collared free‐ranging badgers (that were *M. bovis* negative) and tracking cattle grazing regimes by farmers recording cattle locations and movements. The specific objective was to quantify badger and cattle space use with farm management to production systems, land use, cattle life stages, livestock stocking densities, fragmentation of grazing lands, field size, as well as the presence of badger setts and latrines.

## 2 Materials and Methods

### 2.1 Study Site

A 5‐year Test and Vaccinate or Remove (TVR) badger intervention study, conducted by the Department of Agriculture, Environment and Rural Affairs (DAERA) (O'Hagan et al. 2021), involved the annual deployment of GPS (Global Positioning System) collars on a subset of badgers within a population, which tested bTB negative before release (O'Hagan et al. 2021). This area consists of a rolling hill (drumlin type) landscape. Within the TVR project, badgers that were cage‐captured annually in locations > 1 km from the study boundary during July–October were assessed for their suitability for wearing a GPS collar. Approximately 40 GPS collars (37 collars in Year 1; 36 collars in Year 2; 41 collars in Year 3; 35 collars in Year 4) were applied to badgers (that were bTB test negative) annually with the aim of targeting one adult male and one adult female per social group (Menzies et al. 2021). The badger population estimate across the TVR area was estimated to be 560 badgers with capture rates of between 48%–61% annually (Menzies et al. 2021).

Field surveys to identify badger setts were undertaken prior and during the study (following the methods of Reid et al. (Reid et al. 2011)), mapping sett locations using ArcMap 10.7.1 (ESRI, California, USA). The land use of each land parcel within the study area was captured and categorised as: fodder (grass grown for silage), improved grass (cattle pasture), rough grazing, ‘other’ cattle grazed land (often used to store fodder bails, outdoor machinery and other farm areas), lay (i.e., grass/crop rotation), cereal crop, potatoes and seminatural cover type (including woodland). DAERA provided anonymised land parcel and land use data covering the TVR Project area.

The current study was undertaken within a 54 km^2^ subsection of the TVR study site (Figure 2) that included 25 focal farms that agreed to take part in the study and whose land use was largely representative of the surrounding landscape. The study site had a high density of cattle; the average cattle density was 2.5 cattle/ha for dairy farms and 1.5 cattle/ha for beef farms. It was within a ‘bTB hotspot’ with herd outbreaks recorded for several years (Wright et al. 2015; DAERA 2018; Milne et al. 2019). Cattle production systems were dairy or beef, which were typical of the wider landscape. Many farms in Ireland use fragmented land parcels, which include the letting/purchase of land as single fields, small aggregations of fields, or land parcel patches or strips (Milne et al. 2022). Thus, some farms were fragmented with fields belonging either to the home farm or outlying fragments some distance from the home farm, where cattle movements between such fragments are a recognised risk factor for bTB transmission (Milne et al. 2022).

### 2.2 Cattle Locations

Twenty‐five farmers (11 dairy and 14 beef farms) were given an individual record book with maps of their own farms, with each field given a Unique ID, which was linked to its spatial location. Farmers recorded, for every batch of grazing cattle, the Unique ID of the field and the start and finish dates the field was grazed. Batch life stages were recorded as: bullocks, calves, cows, and heifers. Data were recorded daily from May to November 2016. A detailed description of the methods used to record cattle locations is provided by Campbell et al. (2020).

### 2.3 Badger Locations

GPS locational data were available for 35 collared bTB test‐negative badgers whose ranging behaviour spanned the study farms for which cattle‐grazing regimes were recorded (Tellus light GPS collar (Followit Wildlife, Lindesberg, Sweden; weight 240 g; onboard storage and global system for mobile communication (GSM) download)). GPS collars relayed locations when badgers were active above ground, with a single location recorded every hour (from 9 pm—4 am GMT; based on expert advice). Due to licence restrictions, collars could only be deployed from July, and their placement was stopped in October for each of 2 years. The majority of GPS collars deployed on badgers during the first year had stopped transmitting before the start of the next grazing season the following May (O'Hagan et al. 2021).

Only fields that had the possibility of use by a collared badger were retained in analyses. The fields within a badger's home range (based on its 95% fixed kernel; (O'Hagan et al. 2021)) were included. These fields were only included for the dates in which badger collars were known to be working. The badger GPS data in this study only reflect badger presence in a location rather than the preference or proportion of time spent in the area.

### 2.4 Statistical Analysis

For cattle field use, a ‘field‐day’ was defined as the use of a unique field by a unique group of cattle on a specific date and was recorded as a dichotomous variable for each field (presence/absence). Similarly, for badger field use, a ‘field‐day’ was defined as the use of a unique field by a unique badger on a specific date and was recorded as a dichotomous variable for each field (presence/absence). Multiple badger GPS locations for the same individual badger in the same field on the same day were removed from analysis to avoid pseudo‐replication. The overlap between badger ranges (the distribution of locational fixes) and fields within the study farms for which cattle data were available were mapped.

Badger field use (presence/absence) was analysed, where the unit of variance was the field‐day (*n* = 56,202 field‐days), using a Generalised Linear Mixed Model (GLMM) with a binomial distribution fitting FieldID nested within FarmID as a Random Factor to account for temporal nonindependence (i.e., multiple observations per field throughout the grazing season interacting with spatial nonindependence (fields belonging to the same farm) and thus largely occupying similar space under similar management). The number of badgers available to use a field each day was incorporated into the model using the ‘weights’ function. Date was fitted as a covariate to test for any temporal trend in badger activity (independent of any pattern in data density) throughout the study period. Fixed factors fitted included: production system, field size, cattle presence (presence or absence), badger sett and latrine presence, land use category and land fragmentation (contiguous home farm or outlying land parcels). Field types retained for analysis were limited to cattle‐grazed land use only (i.e., fodder, rough grazing, improved pasture and ‘other’ cattle‐related land uses). To examine the effect of the presence of cattle actively grazing, several interactions were also fitted including Field size*Cattle presence, Production*Cattle presence, and Land use*Cattle presence. A second GLMM was fitted for badger field use but restricted to grazed fields only (*n* = 12,347 field‐days) to allow two factors that could not be fitted within the global model (due to missing data for ungrazed fields); they were livestock stocking density (fitted as a covariate) and batch life stage (fitted as a fixed factor).

To evaluate whether individual badger behaviour (BadgerID) affected the presence or absence of badgers in fields, individual analyses using Generalised Linear Models (GLMs) were performed, incorporating each explanatory variable.

Statistical analysis and plots were produced using R version 3.6.3 (R Core Team 2018). The packages ‘lme4’, ‘ggplot2’ and ‘ggpuber’ were used to produce the models and graphics. Map locations were created in Arc GIS Pro (ESRI Systems, USA).

### 2.5 Ethics Statement

Badgers are a legally protected species covered by the Wildlife Order (Northern Ireland) 1985 as amended by the Wildlife and Natural Environment Act (Northern Ireland) 2011. The TVR Project operated under the Animals (Scientific Procedures) Act 1986 (as amended); ASPA Licence Number 2767. Licences were also obtained from the Northern Ireland Environment Agency (NIEA) to allow the capture, sampling, collaring and removal of badgers.

## 3 Results

A total of 18 farms (72%) completed grazing records that were retained for analysis. Although the minority of farms (*n* = 7) were dairy herds, they had more individual fields covering 10% more area than beef farms (537 v 484 ha) and accounted for the majority of field‐days (55%) recorded throughout the study period (Table 1A,B). Thus, even though dairy and beef fields were similarly frequented by grazing cattle (21% and 23% of field‐days, respectively), dairy accounted for a larger absolute number of grazed field‐days (*n* = 6366) than beef production (*n* = 5981) (Table 1A). Further detailed information on the cattle grazing data for the grazing season has been published elsewhere (Campbell et al. 2020). The number of field‐days each batch of cattle contributed to the data is shown in Table 1C.

**TABLE 1:** (A)Area of farms (hectares) and number of field‐days for each study farm (categorised by production system) throughout the study and the number grazed by cattle, May–November 2016, Test, Vaccinate or Remove study area, County Down, Northern Ireland. (b) Summary of farm attributes (area and herd size), May–November 2016, Test, Vaccinate or Remove study area, County Down, Northern Ireland. (C) Summary data of cattle batches, May–November 2016, Test, Vaccinate or Remove study area, County Down, Northern Ireland.

(A)				
Production type	Farm number	Hectares	Number of field‐days (%)	
			Cattle grazed	All field‐days
Dairy	1	20.2	505 (7.9)	2821 (9.2)
	2	64.3	421 (6.6)	2616 (8.5)
	3	119.0	430 (6.8)	5311 (17.4)
	4	140.5	1943 (30.5)	10,109 (33.0)
	5	63.2	212 (3.3)	1542 (5.0)
	6	59.7	660 (10.4)	2995 (9.8)
	7	70.4	2195 (34.5)	5214 (17.0)
	Subtotal	537.3	6366 (20.8)	30,608 (54.5)
Beef	8	42.0	817 (13.7)	2152 (8.4)
	9	34.6	171 (2.9)	1618 (6.3)
	10	54.8	625 (10.4)	2021 (7.9)
	11	14.0	79 (1.3)	256 (1.0)
	12	44.6	621 (10.4)	4268 (16.7)
	13	10.8	172 (2.9)	456 (1.8)
	14	38.8	1392 (23.3)	4121 (16.1)
	15	32.7	21 (0.4)	472 (1.8)
	16	23.4	168 (2.8)	1021 (4.0)
	17	111.8	63 (1.1)	4417 (17.3)
	18	77.0	1852 (31.0)	4792 (18.7)
	Subtotal	484.5	5981 (23.4)	25,594 (45.5)
	Total	1021.8	12,347	56,202

(B)			
Attribute	Beef	Dairy	Both
Median farm size [ha]	39 (95% CI 23–55, range 11–112)	64 (95% CI 60–119, range 20–140)	50 (95% CI 33–70, range 11–140)
Median herd size (number of cattle)	43 (95% CI 30–80, range 11–114)	168 (95% CI 123–240, range 65–266)	74 (95% CI 36–151, range 11–266)
Number of fields/farm	31 (95% CI 17–38, range 8–84)	40 (95% CI 29–63, range 25–101)	34 (95% CI 25–43, range 8–101)

(C)			
Batch type	Beef	Dairy	Both
Bullocks	3789	1733	5522
Calves	226	1136	1362
Cows	500	1676	2176
Heifers	1466	1821	3287
Total	5981	6366	12,347

**FIGURE 1:**
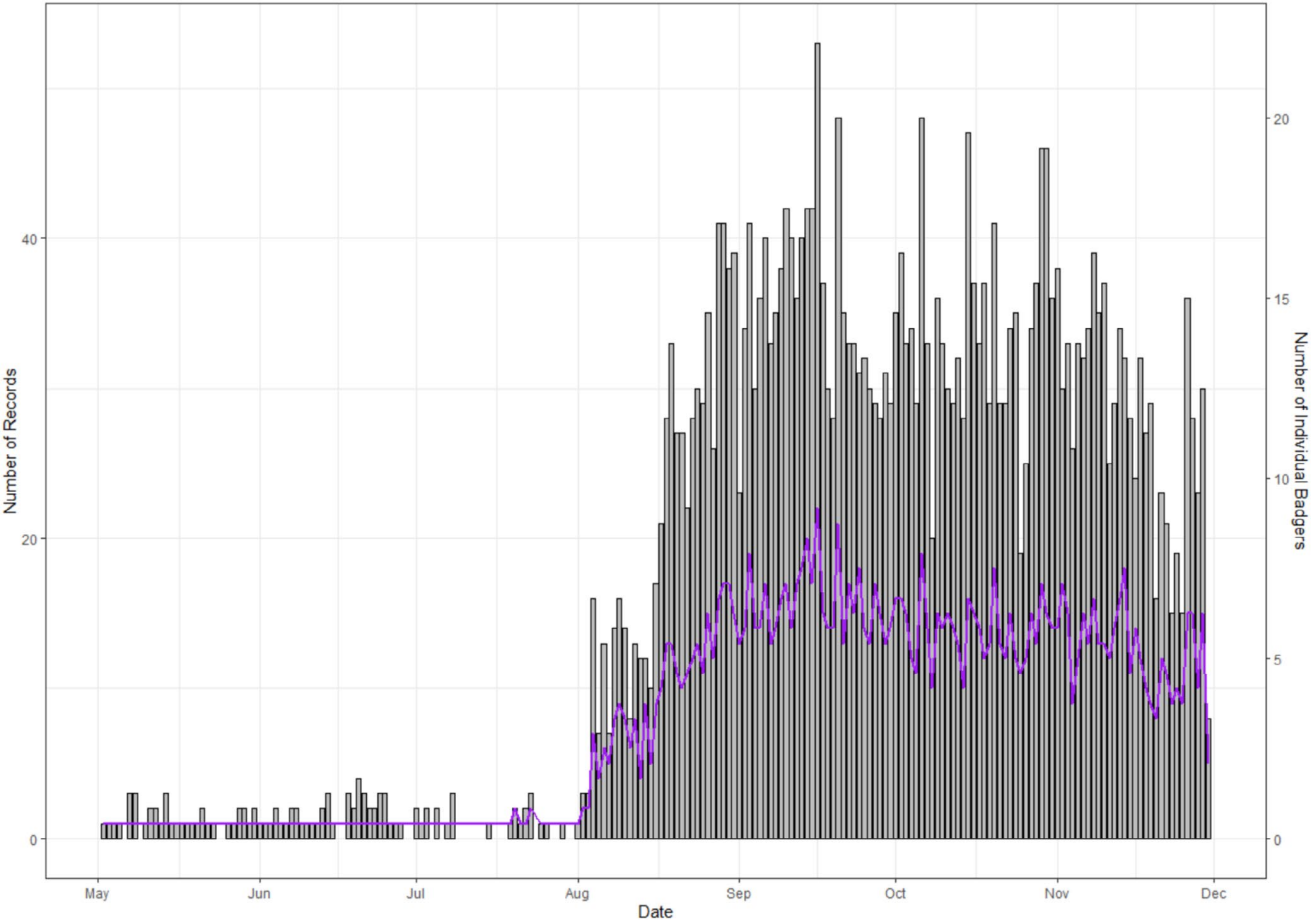
Temporal pattern in badger field‐days (bar chart) throughout the grazing season (May–November) showing variation in data density over the study period. With the number of badgers contributing to each day's records (line graph).

The number of days that fields were used by badgers varied substantially and increased dramatically during the latter half of the study period (Figure 1). Patterns in data density over time were largely contributed to by the accumulation of GPS‐collared badgers throughout the study period as more badgers were recruited into the study over the summer period.

**FIGURE 2:**
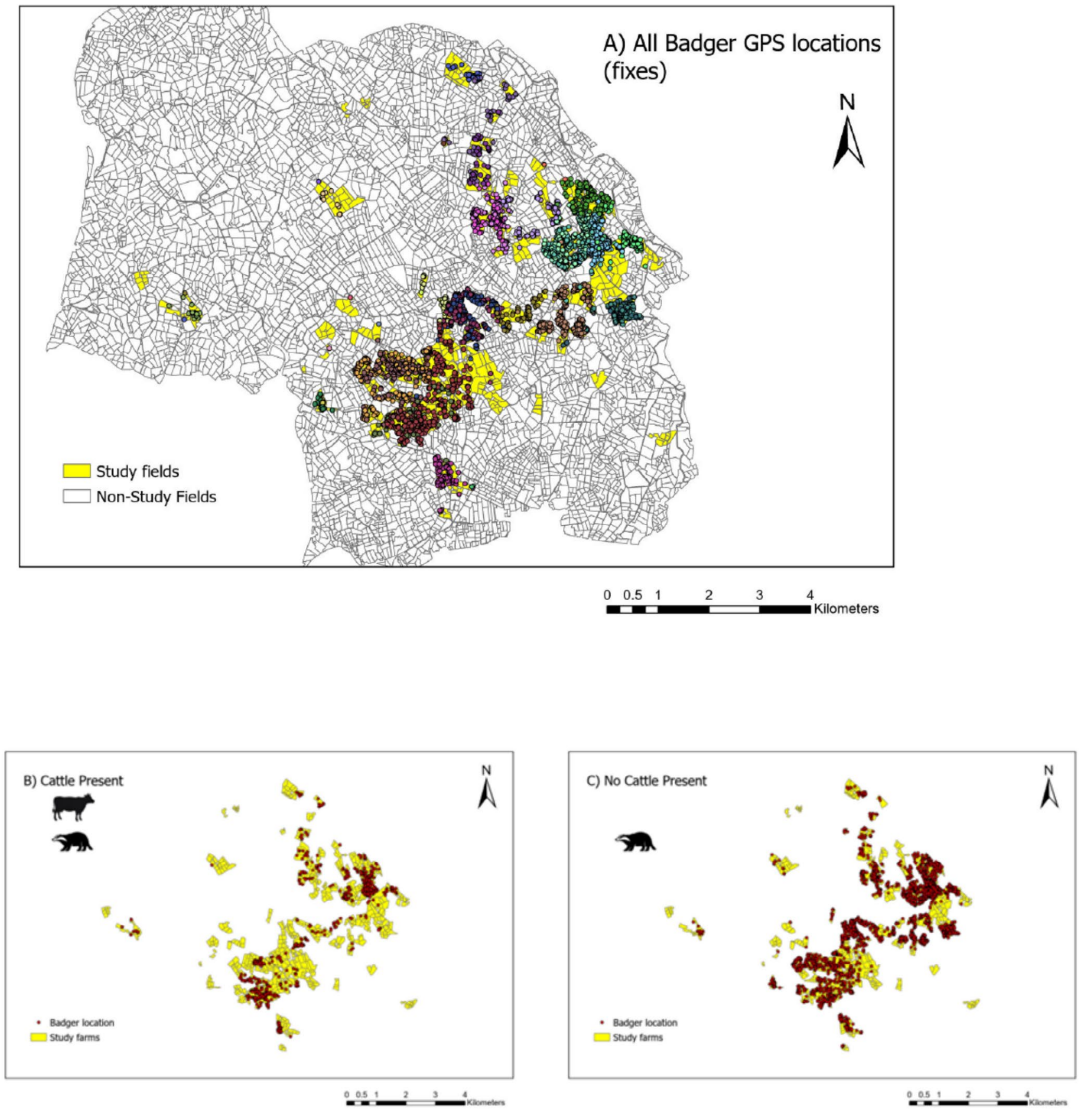
(A) Badger GPS locations (dots) with each colour representing an individual badger (*n* = 35) and the study fields within 18 study farms (coloured yellow) within the wider landscape for context. (B) Badger locations (red dots) within fields with cattle present and (C) fields without cattle present. NB: As the unit of variance for analysis was the field‐day (*n* = 56,202), cattle can be present in a field on some days and not on other days, similarly to badgers.

There were 3738 GPS locations for unique field‐days for 35 individual badgers (mean location fixes/badger 107.1, SE 19.5). Badger ranges spanned a median of 3 farms (95% CI 2–4, range 1–9). A total of 25 badgers (71%) were located in the same field as grazing cattle on at least one field‐day, with a median of 18 such occurrences (95% CI 10–39, range 1–124) throughout the grazing season (Figure 2). Ten badgers (29%) never used a field when it was being grazed by cattle. In total, badgers co‐occurred with grazing cattle on 20% of field days (*n* = 733). No significant relationships were found between individual badgers and their preference for using fields with any of the explanatory variables.

**FIGURE 3:**
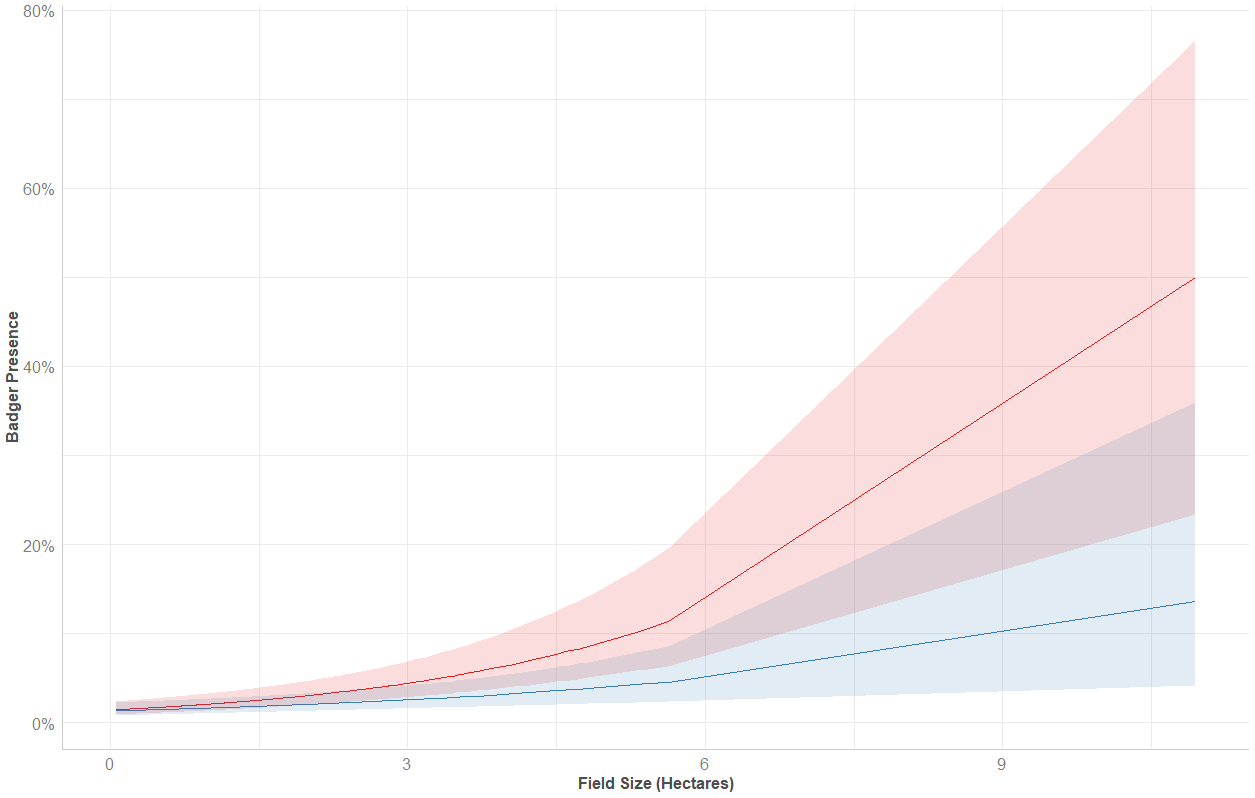
Relationship between field size (hectares) and badger presence where cattle are present (blue) or absent (red) with the solid lines showing the point estimates and the shaded areas indicating the 95% confidence intervals.

Badger field use was significantly affected by cattle presence (Table 2A). Badgers were significantly more likely to use fields of dairy farms. When grazed by cattle, fields owned by beef farms were less likely to be used by badgers. Badgers seemed to be more likely to be found in fields grazed by dairy cattle than when dairy fields were ungrazed. Badger field use was more likely in fields that contained a badger sett (use was unaffected by the presence of badger latrines and land fragmentation). There was also a trend suggesting that land‐use type influenced badgers field use, with fodder and rough grazing used more than improved pasture, with ‘other’ cattle‐related land use utilised least (Table 2A). Badgers were significantly less likely to be recorded in smaller fields (< 4.5 ha) with a trend to be recorded in the larger fields, especially when ungrazed by cattle (Figure 3). There was no temporal trend in badger activity throughout the study period.

**TABLE 2:** Best fitting GLMM results, where field (FarmID (FieldID)) was fitted as a nested random factor and the number of available badgers per field‐day weighted for (A) badger field use across all fields (*n* = 56,202 field‐days) and (B) within cattle grazed fields only (*n* = 12,347 field‐days).

Variable	*χ* ^2^	df	β ± s.e.	95% CI (Lower, upper)	*p*
(a) Badger field use—all fields					
Field size (Hectares)	33.8	1	0.388 ± 0.061	0.268, 0.509	< 0.001
Cattle presence*Production	32.0	1	0.609 ± 0.108	0.398, 0.820	< 0.001
Cattle presence*Field size	26.2	1	−0.163 ± 0.032	−0.226, −0.101	< 0.001
Cattle presence	11.9	1	−0.069 ± 0.115	−0.295, 0.157	< 0.001
Land use	9.3	3	Factorial		0.025
Improved pasture v fodder			−0.289 ± 0.177	−0.635, 0.058	0.102
Rough grazing v fodder			−0.017 ± 0.213	−0.436, 0.401	0.935
Other v fodder			−0.654 ± 0.273	−1.189, −0.118	0.017
Sett presence	3.3	1	0.604 ± 0.331	−0.045, 1.253	0.068
Production	1.7	1	0.220 ± 0.262	−0.295, 0.734	0.191
(b) Badger field use—grazed fields/cattle present					
Batch	42.9	3			< 0.001
Calves v bullocks			−1.567 ± 0.290	−2.135, −0.994	< 0.001
Cows v bullocks			−0.542 ± 0.154	−0.845, −0.240	< 0.001
Heifers v bullocks			−0.750 ± 0.153	−1.050, −0.451	< 0.001
Hectares	9.5	1	0.313 ± 0.102	0.114, 0.512	0.002
Fragmented	7.9	1	−0.715 ± 0.254	−1.212, −0.218	0.005
Production	7.5	1	0.935 ± 0.342	0.265, 1.606	0.006
Stocking density	6.0	1	−0.022 ± 0.009	−0.040, −0.004	0.014

The model restricted to grazed fields suggested that badger field use was affected by livestock stocking density but varied significantly between batch life stages (Table 2B) with badgers more likely to use fields with bullocks present than any other life stage. This is even more evident in fields with dairy bullocks, as the badger predicted probability in fields containing dairy bullocks was ~7% compared with the next highest batch (dairy cows) at ~4% predicted probability.

## 4 Discussion

Overall, the study found a complex relationship between badger and cattle space use, with evidence to suggest that field size and configuration, related to production types, and potentially modulated by badger aversive behaviour towards cattle presence, all contribute to the patterns of interspecies indirect contacts. Individual badgers were recorded across multiple farms, which could be a risk in the transmission of *M. bovis* to farms that were previously test negative.

The herd keeper response rate in the current study was very good, 18/25 farms (72%), especially given the onerous weekly data recording commitment over a whole grazing season that was required. Moreover, the cattle farm demographics of the respondents also broadly matched those recorded at the national level (DAERA 2017). However, the badger monitoring component of this project mostly covered the period of August–November (inclusive) due to the constraints associated with GPS collaring of the badgers, and the results therefore should be considered to mainly relate to that time period (O'Hagan et al. 2021). Badgers in this study were also required to test negative for *M. bovis* infection (Menzies et al. 2021) so any differences in the ranging behaviour of *M. bovis* test‐positive badgers would not have been revealed in this study (Vicente et al. 2007; Cheeseman and Mallinson 1981; Garnett et al. 2005; Böhm et al. 2009). Investigations of GPS‐collared badgers within the TVR area, including during year 1 when no badgers were actively removed, showed that there was no significant difference in the home ranges of the study badgers after *M. bovis* positive badgers were removed from the population (Redpath et al. 2023; O'Hagan et al. 2021; Allen et al. 2022). This provides strong circumstantial evidence that the badger movements recorded in this subset were not affected by the TVR intervention.

Collared badgers were observed on all the study farms during the grazing season. While the majority of badgers (71%) were observed to co‐occur in the same field as cattle, this occurred on 20% of field‐days. This finding concurs with other studies that indicate that badgers do tend to avoid temporal and spatial close proximity to cattle (Drewe et al. 2013; Scantlebury et al. 2004; Woodroffe et al. 2016; Garnett et al. 2005, 2002; Mullen et al. 2013). However, indirect spatial sharing does provide an opportunity for interspecies *M. bovis* transmission given the ability of the organism to survive in the environment (Rodríguez‐Hernández et al. 2016; Allen et al. 2021; Woodroffe et al. 2021), which has been found to be important when modelling spatial variation in transmission between cattle and badgers (Chang et al. 2023). This concept has also been reported for pathogens other than *M. bovis* (Yang et al. 2021). Furthermore, the observed badger ranging behaviour occurring over grazed fields on multiple farms illustrates the potential for increased exposure to *M. bovis* across different herds. If indirect transmission between species does occur, visiting multiple farms increases the potential of a bTB‐infected badger depositing *M. bovis* bacilli, creating contaminated fomites within reach of multiple cattle herds or being exposed to cattle‐deposited fomites, increasing the chances of indirect disease transmission where badgers may transfer disease between neighbouring herds (Corner et al. 2011; Gormley and Corner 2018). The present study illuminated these potential exposures due to the proximity of study farms to each other and the recorded badger locations in this landscape (Figure 2). The reverse is also valid, in that such ranging behaviour also exposes badgers to the potential to encounter bTB infected cattle and their contaminated environment. This is compounded by the degree of farm fragmentation in Northern Ireland, which increases the chances of badgers spatially interacting with cattle from multiple herds (Milne et al. 2022; Doyle et al. 2023).

There was some interindividual variation with a minority of badgers (29%) never co‐occurring with cattle; however, this individual variation was not statistically significant. Therefore, potential badger–cattle indirect bTB infection risk may vary due to individual badger field use behaviour. For example, badgers with very advanced *M. bovis* infection have been found to be more likely to exhibit behaviours that can increase their exposure, such as roaming further and being ‘bolder’ and more likely to enter farmyards and forage closer to farms than noninfected individuals (Vicente et al. 2007; Cheeseman and Mallinson 1981; Garnett et al. 2005). It is currently unclear whether such ‘bold individuals’ are associated with increased risk of interspecies *M. bovis* transmission. The badgers included in this study were required to test bTB test‐negative before a GPS collar was deployed. Therefore, this study may have underestimated ‘bolder’ behaviour of badgers with advanced *M. bovis* infection (Vicente et al. 2007; Cheeseman and Mallinson 1981; Garnett et al. 2005). However, we consider that our study probably provides a good approximation of badgers, including those with subclinical *M. bovis* infection. Furthermore, studies of badger social networks suggest some individuals are more connected than other badgers and with co‐occurring cattle both within and between species and hence may have been more likely to be involved in *M. bovis* transmission (Böhm et al. 2009).

The tendency for badgers to avoid fields actively grazed by cattle is reflected in the findings from other studies (Drewe et al. 2013; Woodroffe et al. 2016; Böhm et al. 2009; Mullen et al. 2013; O'Mahony 2015). This avoidance may in part be due to the mere physical size of cattle, along with their inquisitive nature. Furthermore, the different food sources utilised by the two species (cattle mainly grass and badgers a mixture of plant and animal materials (Byrne et al. 2012)) may naturally separate them spatially (cattle in the main field and badgers at the hedgerow boundary). This separate use of the same spatial areas by the two species circumstantially indicates that indirect interspecies transmission is the most probable infection route between cattle and badgers.

Dairy herds contributed more to the total number of grazing days than beef herds in the study area; this is most likely due to larger dairy land coverage and herd sizes. The median dairy herd size was 168 cattle compared to only 43 cattle in beef herds (Table 1B). Badgers were more likely to graze dairy farms even when cattle were actively grazing. This finding should be interpreted while considering that there were more dairy fields available in this study, but the findings represent a potentially higher risk in dairy herds as they cover larger areas. Irrespective of herd size, dairy farms are at higher risk of contracting bTB, most probably due to their higher stocking density, older animals, increased physiological stress, and higher intraherd contract (Doyle et al. 2014); so it is difficult to assess the impact (if any) that the increased spatial interaction between the species may contribute to this increased risk. Confounding through the interaction of land quality, cattle and badger densities further complicates this interface (Menzies et al. 2019). Dairy cattle may be more likely to be brought supplementary concentrate feed and more likely to be strip grazed (Phillips et al. 2003) than beef animals, which may attract badgers (Scantlebury et al. 2006). It may also be that dairy field improved pastures are more intensely managed for the highest nutritional quality (Patton et al. 2022) while high stocking rates result in heavier grazing and shorter swards, making such fields attractive to foraging badgers. That said, livestock stocking density only produced a small reduction in badger field use, suggesting the presence of cattle may be more important. A recent simulation study using real‐world GIS farm data and GPS‐collared movements by badgers in Ireland also found that across 100 sites, increased incursion risk by roaming badgers was higher for dairy herds than other production types (Murphy et al. 2025). This was related primarily to where dairy herds are established (landscape context), their footprint, and also their relationship to their neighbours (e.g., impacting herd densities) (Murphy et al. 2025).

Table 1C shows the total number of grazing days each batch contributed to the study. Bullocks represented the batch with the most grazing days whereas calves had the least grazing days. The finding that badgers were in calf fields the least may be due to less calf grazing days being available. However, there is the possibility that batch behaviour may play a part in this and should be investigated further. Bullocks were the life stage of beef cattle to have the highest predicted probability of co‐occurring with badgers. It is postulated that bullocks, being heavyweight males, may be less inquisitive and so may leave badgers alone, thus, badgers may avoid bullocks least. Cattle are curious and young animals may be more curious than adults (Krohn 1994) such that calves may approach badgers encouraging avoidance.

Badgers were most likely to occupy improved grass fields (compared to fodder fields, rough grazing and other fields). Improved grass indicates the field has been subject to recent cultivation/reseeding, and pasture length will tend to be intensively managed (frequently grazed and chemically treated). Such farm management practices may well reduce the biodiversity (Plantureux et al. 2005) and relative food abundance for badgers, and, compounded by frequent grazing and less visual cover, will reduce the use of improved grass fields by them.

Larger fields (field size ranged from 0.06 to 10.9 ha) were most likely to be used by badgers, particularly when ungrazed by cattle (Figure 3). The majority of recorded badger locations in larger fields are most likely because larger fields have a greater area and naturally have a higher probability of having a badger present. Further investigation of badger use of different sized fields may be warranted, as there is conflicting evidence in badger use of different sized fields. Larger fields have a greater absolute length of hedgerow, providing a greater abundance of food resources and additionally, hedgerows are favoured for sett locations (Menzies et al. 2019; O'Hagan et al. 2016). However, smaller fields will have a higher perimeter to area ratio, and badgers could therefore spend more time in smaller field types.

Badger setts within a field increased the likelihood of use by badgers. Setts, particularly those accessible by cattle, could be a source of *M. bovis* bacilli and disease transmission risk even if not currently in use by badgers (Courtenay et al. 2006). Sett entrances have been shown to be one of the main sites of badger–cattle interaction (Campbell et al. 2019) with cattle observed nosing directly into the entrance. This could enable aerosol exchange of *M. bovis* droplet nuclei between infected animals of either species (Menzies and Neill 2000). Moreover, *M. bovis* can survive for several weeks in the environment (Rodríguez‐Hernández et al. 2016; Allen et al. 2021). Some badgers with generalised *M. bovis* infection are known to excrete the organism in their urine, which can be deposited on a spoil heap (Corner et al. 2011). Cattle are known to interact with spoil heaps (e.g., head rubbing), which could then disturb any viable *M. bovis* bacilli, thus aerosolising them. Avoiding grazing fields with badger setts is advocated (Scantlebury et al. 2004), or where they cannot be avoided, fencing around setts to prevent direct access by cattle may further reduce contact with contaminated fomites such as spoil heaps.

As fodder and rough grazing fields were used most by badgers, ensuring that cattle are not grazed on silage fields immediately after harvest may also reduce indirect contact, especially at latrines (Ward et al. 2006). Allowing sward length to increase again would encourage cattle to graze away from field boundaries (speed of grass growth can vary depending on time of the year but it should be assessed before allowing cattle access). While predominantly using improved pasture for grazing rather than rough semi‐improved fields would further reduce risk, these suggestions may provide low‐cost, practical strategies by which grazing regimes could be altered to reduce or minimise indirect badger‐cattle contact while cattle are grazing at grass, reducing potential bTB transmission risk for both species. Modelling would suggest that a wildlife reservoir is probably necessary to explain the patterns of *M. bovis* transmission in cattle, with badgers being the most likely candidate (O'Hare et al. 2021) and modulated via the environment depending on uncertainties around persistence (Chang et al. 2023). Other factors such as the test sensitivity of current bovine testing regimes, cattle movement and farm fragmentation may present a much greater risk of *M. bovis* transmission and, therefore, should be prioritised (Doyle et al. 2023, 2014).

The main findings from this study were that badgers tended to avoid fields with cattle present and that they roamed over multiple farms, which could be a risk for transmission of bTB between cattle herds. The relationships between farm type, cattle life stage and field size require further investigation to fully understand the extent of associations found in this study.

This study has given insight into opportunities that agro‐ecosystems can provide for wildlife species and livestock to interact due to the wildlife habitat components that agricultural regions and their adjacent vegetation types can offer to wildlife. Concepts such as One Health, surveillance and environmental management are therefore key in the prevention of inter‐ and intraspecies disease transmission between domesticated animals and wildlife.

## Author Contributions

**Emma L. Holmes:** conceptualization (equal), data curation (lead), formal analysis (lead), methodology (equal), project administration (lead), writing – original draft (lead), writing – review and editing (equal). **Maria J. H. O’Hagan:** conceptualization (supporting), data curation (supporting), writing – review and editing (equal). **Fraser D. Menzies:** conceptualization (equal), supervision (equal), writing – original draft (supporting), writing – review and editing (equal). **Andrew W. Byrne:** conceptualization (equal), supervision (equal), writing – original draft (supporting), writing – review and editing (supporting). **Kathryn R. McBride:** project administration (supporting). **Charles M. McCormick:** project administration (supporting), writing – review and editing (supporting). **David M. Scantlebury:** conceptualization (equal), supervision (equal), writing – original draft (supporting), writing – review and editing (supporting). **Neil Reid:** conceptualization (equal), supervision (equal), writing – original draft (supporting), writing – review and editing (supporting).

## Conflicts of Interest

The authors declare no conflicts of interest.

## Data Availability

The data used to support the findings of this study are made available for review and will be stored in a repository.
